# The do‐it‐yourself movement as a source of innovation in biotechnology – and much more

**DOI:** 10.1111/1751-7915.12715

**Published:** 2017-04-09

**Authors:** Víctor de Lorenzo, Markus Schmidt

**Affiliations:** ^1^ Systems Biology ProgramCentro Nacional de Biotecnología (CNB‐CSIC)28049Cantoblanco‐MadridSpain; ^2^ Biofaction KG1030ViennaAustria

*The best way to have a good idea is to have a lot of ideas*.^1^ This celebrated quote from Linus Pauling summarizes one of the key drivers of innovation. Breakthroughs and major advances in biotechnology (and science in general) can often be traced to specific individuals who had one sparkling thought that did work – out of an ocean of failures and ideas too heretical to share with the establishment of the time. In fact, many feel that both the academic and the industrial Gotha have become veritable obstacles for innovation. In the first case, professional scientific careers depend to a very large extent on publications in high‐impact journals. This frequently makes academic scientists to focus on a limited number of topics likely to result in high impact factor papers. In the best‐case scenario, risky, out‐of‐the‐box research is the privilege of well‐established individuals who do not jeopardize their careers and reputation when they pursue somewhat crazy ideas. But when young scientists face a thematic choice, the panic to failure and ridicule at an early stage of one's academic career often place invisible barriers to creativity and curb the pursuit of new ways to tackle scientific and technological questions. Even if young scientists are brave enough to challenge inherited wisdom and come forward with smart, creative and bold ideas, more often than not they are unable to attract funding and support from senior colleagues (Nicholson, 2012). In industry, the issues are somewhat different, but equally worrisome. In this case, the rampant paranoia about intellectual property (IP) not only inhibits candid discussions among specialists on a given topic (one of the best cradles for new ideas) but it also kicks very inventive minds (who use to abhor any restriction in their thinking) away from the industrial realm. One deplorable consequence of the current IP frame is that patents that operate on specific materials and technologies become actual deterrents of their further development by those not inclined to pay the licences. This makes much of the IP held by industry to be well protected, but ultimately useless. And a wealth of possible discoveries and innovation opportunities may never be born. Another constraint in industry is the fact that innovations do need to fall under the companies’ strategic aims and must have the real potential to bring a return of investment in a reasonable timeframe. Finally, a third vector of such an innovation‐unfriendly landscape that afflicts both academia and industry is the cost of modern biological research. Large investments in equipment, facilities and information management seem to be a must for producing high‐level bioscience and biotechnological breakthroughs. This leaves behind large communities, even whole countries, which may have the talent but lack the money to assemble the facilities and resources necessary to translate ideas into value. This is accompanied by an inevitable brain drain from developing countries to wealthier ones, with the collateral effect that the focus is shifted from Third World to First World problems.

Is there a way out of what appears to be an insurmountable scenario for free‐minded innovation? How can we ensure that ideas worth to pursue are not lost in the whirlpool of academic tracks, IP obsessions, corporate interests and dearth of money? The last few years have witnessed a few initiatives to mitigate such state of affairs. One involves the growing popularity of open source and open access, which ambition the free availability of information and materials resulting from research carried out under public funding. Revealing cases in this regard include the recent release by NASA of a software catalogue, aimed at granting the public free access to technologies for earthly applications^2^. The obligation to publish in open access all papers resulting from research funded by, for example, the US’ NIH or the EU's ERC and the success of repositories of genetic constructs such as Addgene^3^ or SEVA^4^ are indicators of the same trend as well. A second tendency is what one could call low‐cost high‐tech or *frugal technology*. In this case, the idea is to produce with cheap materials a suite of instruments whose price tag is generally prohibitive, thus making them available to more potential users inside or outside the academic or the industrial frame. One archetypal example in this direction is the work of Manu Prakash at Stanford,^5^ which pursues design solutions for extremely resource‐constrained settings, especially in the field of global health. Two items resulting from Prakash's work, the *foldscope* (Cybulski *et al*., 2014) and the *manual paper centrifuge* (Bhamla *et al*., 2017), made headlines recently, as they combine sound engineering with amazingly cheap production and ease of use. In the same path, it is possible to find second‐hand laboratory equipment for ridiculously low prices in eBay. And a large number of instruments including quite complex ones like atomic force microscopes^6^ and environmental sensors^7^ have become available at very low cost that allow setting of research laboratories outside the academic territory. The onset and spreading of cheap 3D printing also helps in making biological research devices available to a much larger number of people beyond the habitual control of universities and industries.

In this context, it is little surprising that the last few years have seen a growing number of people who have embraced what is commonly labelled as *biohacking*,* amateur* or *do‐it‐yourself biology* (DIYBio). The term *hacking* was coined by computer and electronic engineering students at MIT who, in a playful competition, demonstrated their ingenuity in rewiring the control circuit of a model railroading system (Levy, 1984). While initially it refers to any type of clever solution,^8^ hacking was eventually also done in computer systems for which the term became publicly famous. Contrary to the popular belief that hackers carry out criminal activities, their culture may be described by four interrelated goals: (i) to investigate a subject for its own sake, (ii) to engage in non‐destructive mischief, (iii) to do something out of the ordinary or clandestine and (iv) to crack the inaccessible. Over the years, students and engineers, first at MIT and then all over, have maintained hacking as a self‐driven, problem‐solving state of mind that autonomously searches and identifies challenges to be resolved. The motivation of hackers can be manifold, ranging from the need to solve a particular technical problem, to a mild form of bragging over one's technical capabilities, to humorous motives (including lampooning authorities), to support freethinking in a world of heteronomous rules and power structures. Given the history of hacking at MIT, it became natural that biohacking also emerged from computer engineers in the same location, such as Tom Knight, who became interested in improving genetic engineering at the turn of the millennium. Knight also co‐founded the synthetic biology iGEM competition in 2004, an international student contest with the aim to construct novel engineered life forms for constructive purposes. iGEM^9^ is based on standard biological parts, or BioBricks, that are conceptually modelled after electronic parts to allow for an easier and more efficient genetic engineering. Such BioBricks are made available to a wide community of (mostly young) users with little concern about possible IP issues, just by disclaiming carrier liability. Some of the earliest student participants in iGEM were later among the pioneers in the DIYBio/biohacker scene first in the United States, then in Europe and elsewhere. Is this altogether new? It is important to state that to an extent, humans have always been *biohackers:* farmers, brewers, chefs, etc. have done nothing but hacking Nature, a trend that continues to this day.^10^ Prominent earlier members of this movement at the MIT founded later Ginkgo Bioworks,^11^ a synthetic biology company now worth >150 Mo USD. Note that information on DIY activities and achievements rarely appears in academic journals; the free‐flowing Internet has become instead the favourite channel to this end.

Biohacking, broadly described, aims to understand, redesign and produce new forms of life – and/or other endeavours in the biological realm and the tools required to do so, but not necessarily through the established channels, and often at odds with them. The onset of a larger DIYBio movement around the World in the last 10 years or so has led to a variety of descriptions, analyses and interpretation of what DIYBio and biohacking really are, including gross exaggeration and belittling (Seyfried *et al*., 2014). A rather negative view is the portrayal of a potentially subversive community that is a biosecurity (bioterrorism) risk. Others pointed out that the diffusion and democratization of genetic engineering tools outside of traditional academic and industrial laboratories would raise safety concerns and accidents (Schmidt, 2008). DIYBio, however, is also seen as a great way to teach students the basics of biology and bioengineering in a fun and exciting way, and many of the community laboratories around the world seem to follow this approach (e.g. Genspace in New York^12^ or The Waag Society in the Netherlands^13^). DIYBio is also allowing for unusual research projects – not devoid provocation, such as the yoghurt made from vaginal bacteria.^14^ What is indeed special in the DIYBio world is the collaborative attempt of the community to provide the tools and methods to engineer biology in way that is transparent (open source) and affordable to people with a small budget. Landrain *et al*. (2013), for example, listed a number of machines and tools found in every biolaboratory together with its regular price tag and then present hacked tools and devices that usually cost only a fraction and in most cases work almost as good as the commercial kits/tools.^15^ A recent example of making personal laboratory automation accessible to more people is the OpenDrop device by GaudiLabs^16^ to handle small amounts of fluidics. Even sophisticated techniques such as gene editing with CRISPR can now be done in one's backyard with available gene engineering kits. Yet, it remains to be seen whether the start‐ups who sell these kits are able to provide sufficient quality assurance for their products.

Another approach that tries to tackle the same challenge, namely the prohibitive costs of equipping and maintaining a full‐fledged research laboratory, can be seen in the sharing of equipment for (potential) start‐up companies in the biotech sector. As laid out by Sia and Owens (2015), more laboratories of this type are established around the world, fostering the creation of biotech companies that keep financial hurdles low for prospective businesses. Several start‐ups have directly addressed the growing community of biohackers and biodesigners, providing desktop machines to engineer bacteria with a modular plasmid system. Examples include The Microbial Design Studio,^17^ The Bento Lab^18^ and the The Amino Lab.^19^ But innovation in DIYBio is not only restricted to providing cheap access to useful machines, either in incubator farms or at home. It is also a different ethos of sharing and global tinkering that spurs innovative ideas (Kera, 2014). Whatever the reason for innovation might be, more and more of them are being noticed by the Venture Capital World. Dedicated incubators have been established that especially support biotech start‐ups, such as the outstanding RebelBio incubator^20^ (previously called IndieBio) that has already lifted up more than two dozen biotech start‐ups with many more to come.

Will DIYBio change the game for biotech innovation? This remains to be seen, but certainly the trend towards a broader involvement of more players in the design and fabrication of new business ideas will continue in the upcoming future – which should cause more joy than concern. These developments can also bring a fresh air to the oppressive climate of IP rights that, as argued above, limit so many possibilities. Note also that the freedom enjoyed by the DIYBio community will necessarily cause noise, as any frontier activity does. But the return in terms of creativity and new ideas for the sake of the biotechnological sector will be phenomenal. Let us thus celebrate the promise that this reaching out to new talent and innovation will be a powerful new motor of enterprise and job creation.

## Notes

1
http://oregonstate.edu/dept/Special_Collections/subpages/ahp/1995symposium/crick.html

2
https://www.nasa.gov/press-release/nasa-releases-software-catalog-granting-the-public-free-access-to-technologies-for

3
https://www.addgene.org

4
http://seva.cnb.csic.es

5
http://web.stanford.edu/group/prakash-lab/cgi-bin/labsite/

6
https://goo.gl/HLP6HM

7
https://hackaday.io/project/13135-sensors-list/log/43776-sensors-list

8
https://hacks.mit.edu/by_year

9
http://igem.org

10
https://www.noisebridge.net/images/4/41/CENTGGNoiseBridgePPT_2.pdf

11
http://www.ginkgobioworks.com

12
https://www.genspace.org

13
http://waag.org/en

14
https://motherboard.vice.com/en_us/article/how-to-make-breakfast-with-your-vagina

15 See also http://www.hackteria.org

16
http://www.gaudi.ch/OpenDrop/?p=17

17
http://www.biorealize.com

18
https://www.bento.bio

19
https://amino.bio

20
https://rebelbio.co
